# VIP Enhances Phagocytosis of Fibrillar Beta-Amyloid by Microglia and Attenuates Amyloid Deposition in the Brain of APP/PS1 Mice

**DOI:** 10.1371/journal.pone.0029790

**Published:** 2012-02-06

**Authors:** Min Song, Jia-xiang Xiong, Yan-yan Wang, Jun Tang, Bo Zhang, Yun Bai

**Affiliations:** 1 Department of Medical Genetics, Third Military Medical University, ChongQing, China; 2 Department of Medical Physiology,Third Military Medical University, ChongQing, China; Virginia Commonwealth University, United States of America

## Abstract

Vasoactive intestinal peptide (VIP) is a multifunctional neuropeptide with demonstrated immunosuppressive and neuroprotective activities. It has been shown to inhibit Amyloid beta (Aβ)-induced neurodegeneration by indirectly suppressing the production and release of a variety of inflammatory and neurotoxic factors by activated microglia. We demonstrated that VIP markedly increased microglial phagocytosis of fibrillar Aβ42 and that this enhanced phagocytotic activity depended on activation of the Protein kinase C (PKC) signaling pathway. In addition, VIP suppressed the release of tumor necrosis factor alpha (TNF-α) and nitric oxide(NO) from microglia activated by combined treatment with fibrillar Aβ42 and low dose interferon-γ (IFN-γ). We utilized an adenovirus-mediated gene delivery method to overexpress VIP constitutively in the hippocampus of APPswPS1 transgenic mice. The Aβ load was significantly reduced in the hippocampus of this animal model of Alzheimer's disease, possibly due to the accumulation and activation of cd11b-immunoactive microglial cells. The modulation of microglial activation, phagocytosis, and secretion by VIP is a promising therapeutic option for the treatment of Alzheimer's disease(AD).

## Introduction

Alzheimer's disease is a progressive neurodegenerative disease characterized by senile plaques, intracellular neurofibrillary tangles, and reactive gliosis involving both microglia and astrocytes [1], [2]. Microglia are derived from mononuclear phagocytes and function as the resident macrophage-like cells of the brain parenchyma. Microglia serve functions similar to other resident macrophages, including phagocytosis, antigen presentation and production of cytokines. Microglia-mediated immune responses play both protective and deleterious roles in the pathogenesis of AD. On one hand, microglia secrete neurotrophic agents and eliminating beta-amyloid through direct phagocytosis. Conversely, microglia release neurotoxic (pro-inflammatory) cytokinase and other factors that can cause neurodegeneration, including NO. Therapeutic strategies that inhibit the secretion of neurotoxins from microglia or enhance microglial phagocytic activity may reduce cerebral Aβ load in mouse models of AD and thus limit neurodegeneration [3]–[6].

Several endogenous brain mechanisms serve to dampen inflammatory responses in the brain. As one group of critical anti-inflammatory agents, neuropeptides maintain the immunological privilege of the central nerve system (CNS) [7]. For example, calcitonin gene-related peptide [8], adrenomedullin [9], neuropeptide Y [10] and somatostatin [11] are anti-inflammatory immune modulators. Several recent studies indicated that vasoactive intestinal peptide can also modulate immune function through G-protein coupled receptors expressed by immune cells and that VIP signaling is an important component of a homeostatic neuroimmune control system [12] [13].Vasoactive intestinal peptide is a 28-amino acid peptide secreted by peptidergic neurons located in all regions of the cerebral cortex, limbic forebrain (septum, amygdala, hippocampus, and stria terminalis) and hypothalamic areas (paraventricular and periventricular nuclei, arcuate nucleus, and anterior and preoptic areas). It is believed that VIP-containing nerve terminals constitute an anatomical link between the CNS and immune system [14]–[16].

Vasoactive intestinal peptide exerts anti-inflammatory effects on microglia by activating two common VIP receptors: VPAC1 and VPAC2. These receptors stimulate adenylate cyclase (AC), which increases intracellular cyclic adenosine monophosphate (cAMP) concentrations and leads to downstream activation of protein kinase A (PKA) and PKA-responsive transcription factors. The release of pro-inflammatory TNF-α, interleukin(IL)-1β, IL-6, NO from bacterial lipopolysaccharide (LPS)-activated primary microglial cultures was marked reduced by 10^−8^ M VIP [17] [18]. Lipopolysaccharide and interferon gamma (IFN-γ)-activated microglia also produced less macrophage inflammatory protein (MIP)-2, MIP-1α, keratinocyte derived chemokine (KC), RANTES (regulated upon activation normal T cell expressed and secreted protein and γ-interferon inducible protein (IP-10) in response to VIP treatment [17] [19]. Thus, VIP stimulation of activated microglia reduces the secretion of a multitude of proinflammatory chemokines and cytokines. Expression of VIP is significantly decreased in the cerebral cortex of aged animals and VIP blockade in younger animals resulted in impaired learning and memory [20]–[21].

In addition, VIP secretion was significant reduced in the insular and angulate cortex of AD patients as measured by radioimmunoassay [22], while the number of neurons expressing VIP was significantly reduced in the suprachiasmatic nucleus of female pre-senile AD patients [23]. It is still unclear, however, whether this reduction was the cause or the result of cortical deterioration and AD-related cognitive decline. A recent study did manage to show that VIP inhibited Aβ-induced neurodegeneration by indirectly inhibiting the production of a battery of inflammatory and neurotoxic agents by activated microglia cells [24], suggesting that a VIP deficit may contribute to AD-associated focal cortical degeneration. Whether VIP treatment to the brain can improve AD pathogenesis has still not been demonstrated. Furthermore, increased phagocytic capacity of microglia after VIP treatment, a possible mechanism to reduce cerebral Aβ load, has also not been directly observed.

The present study investigated the effect of VIP on the phagocytosis of Aβ by microglia and on the secretion of proinflammatory cytokines by microglia in response to Aβ. To demonstrate the beneficial effect of VIP *in vivo*, we overexpressed VIP in transgenic PS1/APP mice and examined whether this treatment could decrease brain Aβ load in this AD model.

## Results

### VIP promote phagocytosis of fibrilized Aβ_1–42_ by activated murine microglial cells

**Figure 1:**
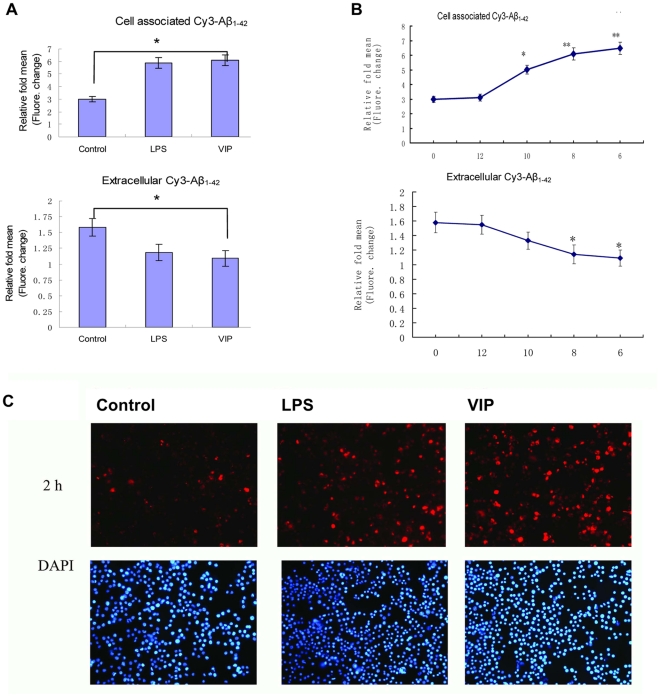
VIP treatment promote microglial phagocytosis of fibrillar Aβ_1–42_. A: Cell supernatants and lysates were analyzed for cell-associated (top panel) and extracellular (bottom panel) Cy3-Aβ_1–42_ using a fluorometer. Data are represented as the relative fold of mean fluorescence change (mean±SD), calculated as the mean fluorescence for each sample at 37°C divided by mean fluorescence at 4°C (n = 5 for each condition presented). One-way ANOVA followed by post-hoc comparison showed a significant between-group difference (*P<0.05, **P<0.01 compared with control). B: Primary microglia cells were stimulated with fibrillar Aβ_1–42_ (500 nM) in the absence or presence of different concentrations of VIP(−logM). Cell supernatants and lysates were analyzed. for cell-associated (right panel) and extracellular (left panel) Cy3-Aβ_1–42_ using a fluorometer (n = 5 for each condition presented). C: Mouse primary microglial cells were seeded in twelve-well tissue-culture plates (2×10^5^/well) and treated with 500 nM Cy3-Aβ_1–42_ in the presence or absence of VIP(10^−8^ M), LPS (100 ng/ml, as positive control). After 2 hr, these cells were washed and fixed (see Materials and Methods). Subsequently, immunofluorescence microscopy examination was performed with a ×10 objective with appropriate filter selection. The up panels show Cy3-Aβ_1–42_ associated with microglial cells under the various treatment conditions. The below panels (DAPI) reveal the presence of the microglia cell as identified by their nuclei.

We first examined the effects of VIP on the phagocytic functions of mouse primary microglial cells. To determine if VIP can modulate microglial uptake of Aβ_1–42_, mouse primary microglial cells were incubated in 500 nM fibrilized-Cy3-Aβ_1–42_ for 2 h in the presence or absence of VIP. Cell supernatants and lysates were analyzed for extracellular and cell-associated Cy3-Aβ_42_ using a fluorometer (Fig. 1A, B). Co-incubation with VIP significantly enhanced microglial phagocytosis of Aβ_1–42_ peptide as evidenced by the increase in cell-associated Cy3-Aβ_42_ fluorescence (Fig. 1A, top panel) and the significant reduction in extracellular Cy3-Aβ_42_ (Fig. 1A, bottom panel). As a control for the non-phagocytic incorporation of Aβ_1–42_ by microglia, microglial cells were incubated at 4°C under the same treatment conditions. As a positive control, Cy3-Aβ_42_ uptake was measured in the presence of the ubiquitous microglial activator LPS; indeed LPS increased microglial phagocytic Cy3-Aβ_42_ uptake (Fig. 1A, top panel) and reduced extracellular Cy3-Aβ_42_ (Fig. 1A, bottom panel, P<0.05). We next assessed the effect of different VIP doses on microglial phagocytosis of fibrilized-Aβ1_-42_. Phagocytotic uptake was dependent on VIP dose, with maximal intracellular Cy3-Aβ_42_ at 10^−6^ M (Fig. 1B, P<0.05). This enhanced Aβ_42_ phagocytosis was verified by a quantitative immunofluorescence assay. Cultures were incubated in fluorescence-tagged Aβ_42_ in the presence and absence of VIP. Cultured microglia exhibited significantly higher intracellular fluorescence when cultured in the presence of VIP (Fig. 1C).

### PKC inhibitors attenuate VIP-enhanced phagocytosis of fibrilized-Aβ_42_ by microglia

**Figure 2:**
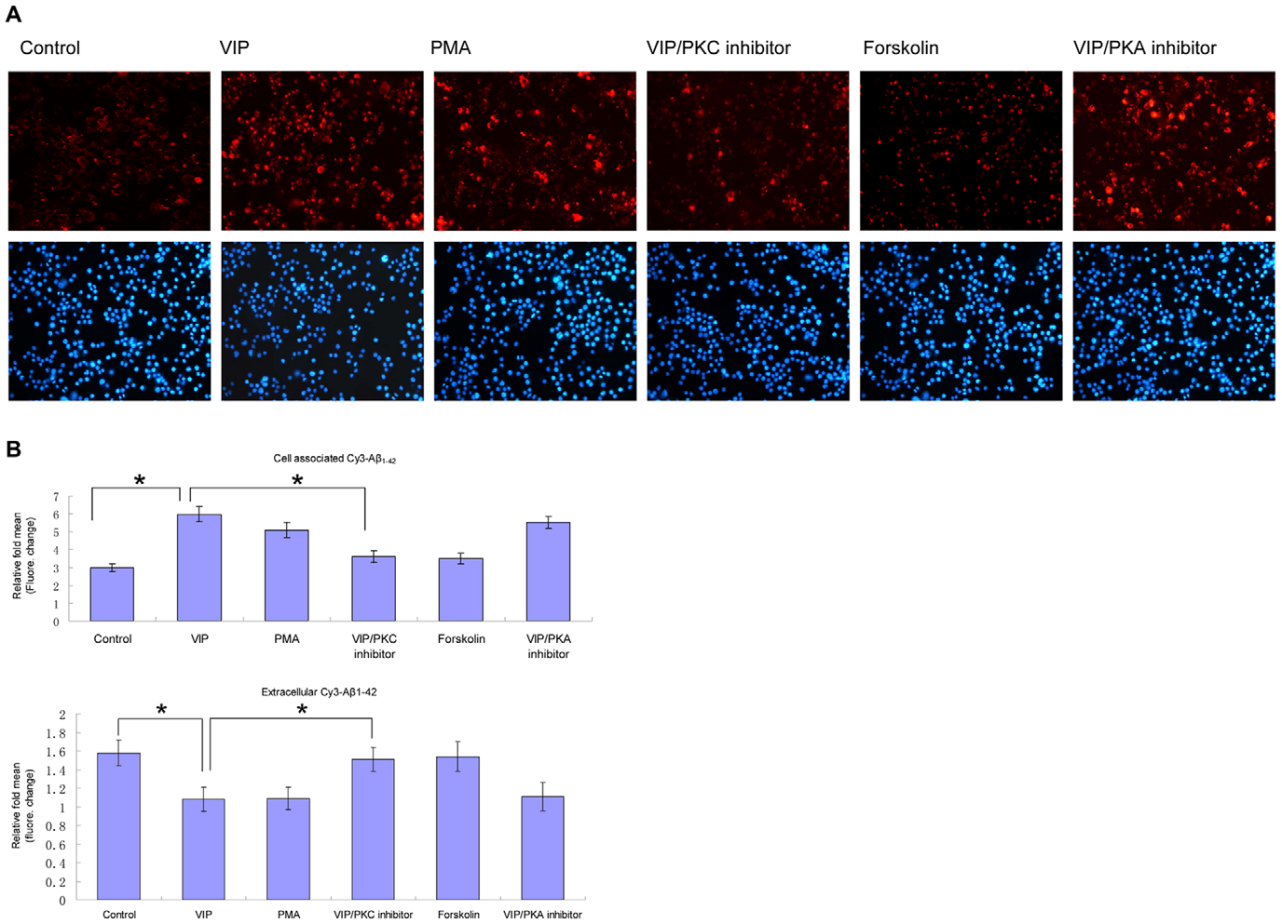
PKC inhibitor attenuates VIP promotion of microglial phagocytosis of fibrillar Aβ_1–42_. *A*: Mouse primary microglial cells were seeded in twelve-well tissue-culture plates (2×10^5^/well) and treated with 500 nM Cy3-Aβ_1–42_ in the presence of VIP(10^−8^ M), PMA (30 nM), Forskolin (10 µM), Myristoylated protein kinase C peptide (50 µM) combined with VIP, PKA inhibitor fragment 14–22 myristoylated trifluoroacetate salt (PKA Inhibitor) (5 µM) combined with VIP. After 2 hr, these cells were washed and fixed (see Materials and Methods). Subsequently, immunofluorescence microscopy examination was performed with a ×10 objective with appropriate filter selection. The up panels show Cy3-Aβ_1–42_ associated with microglial cells under the various treatment conditions. The below panels (DAPI) reveal the presence of the microglia cell as identified by their nuclei. B: Mouse primary microglial cells were seeded in twelve-wells tissue-culture plates (2×10^5^/well) and treated with 500 nM Cy3-Aβ_42_ in the presence or absence of VIP and Myristoylated protein kinase C peptide inhibitor, PMA (PKC pathway activator), cAMP agonists(Forskolin), PKA antagonists(PKA inhibitor fragment 14–22, myristoylated trifluroacetate salt)for 2 hr, Subsequently, Cell supernatants and lysates were analyzed for cell-associated (top panel) and extracellular (bottom panel) Cy3-Aβ_1–42_ using a fluorometer. Data are represented as the relative fold of mean fluorescence change (mean±SD), calculated as the mean fluorescence for each sample at 37°C divided by mean fluorescence at 4°C (n = 5 for each condition presented). One-way ANOVA followed by post-hoc comparison showed a significant between-group difference (*P<0.05, **P<0.01 compared with control).

Delgado et al. found that the stimulatory effect of VIP on freshly isolated resting macrophages was mediated by the PKC signaling pathway [25]. To examine the signal pathways involved in VIP-mediated stimulation of microglial phagocytosis of fibrilized-Aβ_42_, microglial cultures were treated with VIP for 2 hr in the presence of the myristoylated protein kinase C peptide inhibitor or the PKA inhibitor fragment 14–22, myristoylated trifluoroacetate salt. In parallel experiments, microglial cultures were treated with the PKC agonist PMA or the adenyly cyclase/PKA pathway activator forskolin. Forskolin treatment alone did not enhance Cy3-Aβ_42_ phagocytosis compared to controls. In contrast, PKC activation by PMA increase microglia phagocytic activity compared to controls. The PKC pathway inhibitor myristoylated protein kinase C peptide inhibitor abolished the increased phagocytosis of Cy3-Aβ_42_ induced by VIP, while the PKA inhibitor fragment 14–22 had no effect (Fig. 2 A.B). These data suggest that activation of the PKC pathway, but not the PKA pathway, is necessary for the VIP-induced enhancement of Cy3-Aβ_42_ phagocytosis by microglial cells.

### VIP inhibits Aβ_42_-activated TNF-α and NO secretion from murine microglial cells

**Figure 3:**
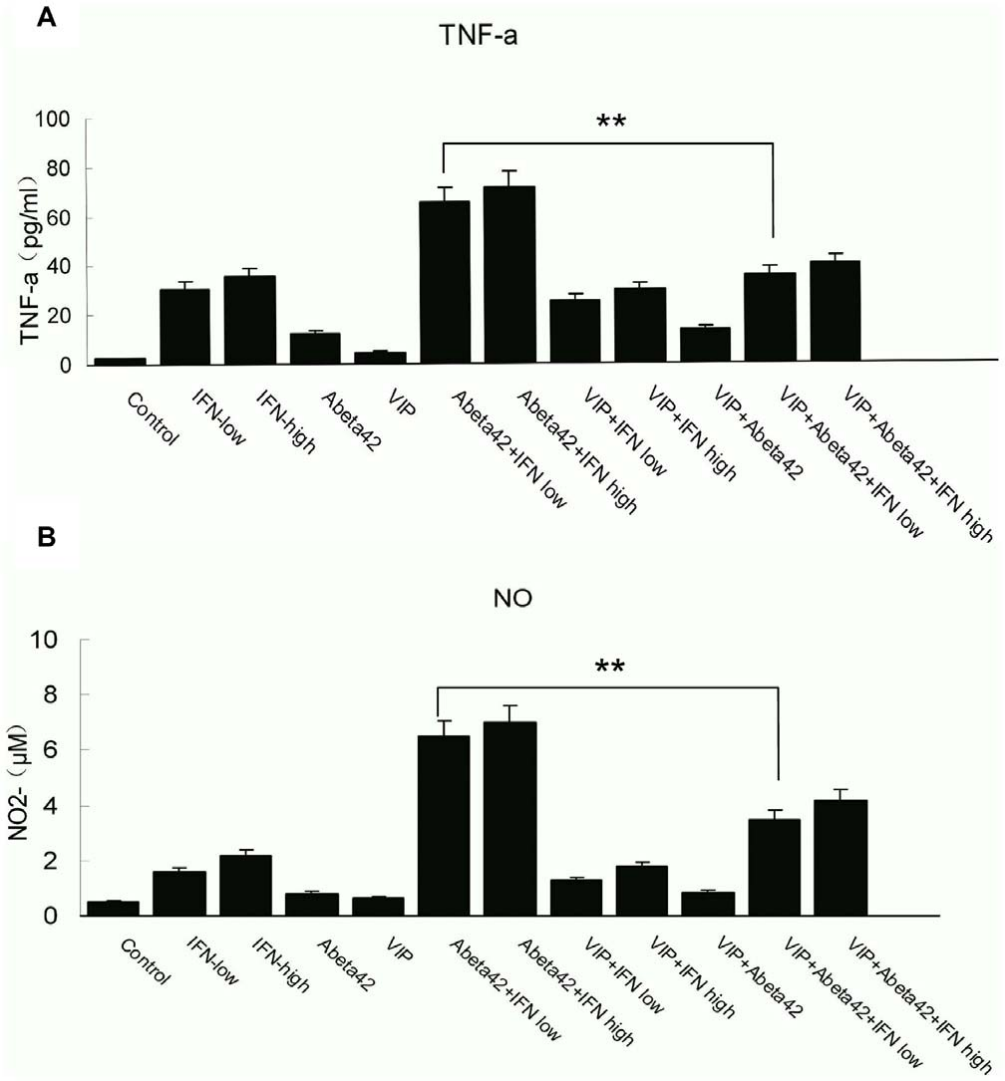
VIP inhibite TNF-α or NO secretion induced by Aβ_1–42_ ((2.5 µM) combined with low dose IFN-γ (10 U/ml) in microglial cells. Microglial cells were plated in 24-well tissue-culture plates at 1×10^5^ cells per well and stimulated for 24 hr with either Aβ_1–42_ (2.5 µM) or a dose range of 10 U/mL IFN-γ (IFN low), 200 U/mL IFN-γ (IFN high)/Aβ_1–42_ (2.5 µM) in the presence or absence of VIP (10^−8^ M). Cell-free supernatants were collected and subjected to TNF-α ELISA or Nitrite Determination Griess Reagent Kit as indicated. Data are the mean±SD of three separate experiments performed in double duplicate. ANOVA and post hoc testing revealed significant differences between different group. **P<0.01 Aβ_1–42_/IFN-γ treated samples plus VIP vs. Aβ_1–42_/IFN-γ treated samples.

Activated microglia can be either neuroprotective or neurodestructive depending on the phase of activation. VIP may promote neuroprotection against Aβ toxicity by promoting phagocytosis. To examine whether VIP also altered the secretion of neurotoxic cytokines or oxidant species, the levels of TNF-α and NO were measured in the supernatant of activated microglia in the presence and absence of VIP. Murine primary microglial cells were activated with either fibrilized Aβ_1–42_ (2.5 µM) alone or fibrilized Aβ_1–42_ combined with low dose IFN-γ (10 U/ml) for 24 h in the absence or presence of VIP. The secretion of TNF-α and NO were analyzed by ELISA. Low dose IFN-γ plus Aβ_1–42_ evoked a robust increase in TNF-α and NO secretion [26]. Treatment with VIP alone (10^−8^ M) did not affect basal TNF-α and NO secretion from resting microglia (Fig. 3A.B) but inhibited TNF-α and NO secretion induced by Aβ plus low dose IFN-γ (Fig. 3A.B, P<0.01). Similar results were obtained in the murine microglial cell line N9 cells (data not shown). Thus, VIP could enhance phagocytosis of Aβ_42_ by activated microglia and inhibit the release of neurotoxic TNF-α and NO by Aβ_42_-activated microglia.

### Adenovirus-mediated VIP expression reduces immunoreactive Aβ deposits and fibrillar Aβ deposits in the hippocampus but not in the neocortex of AD mice

**Figure 4:**
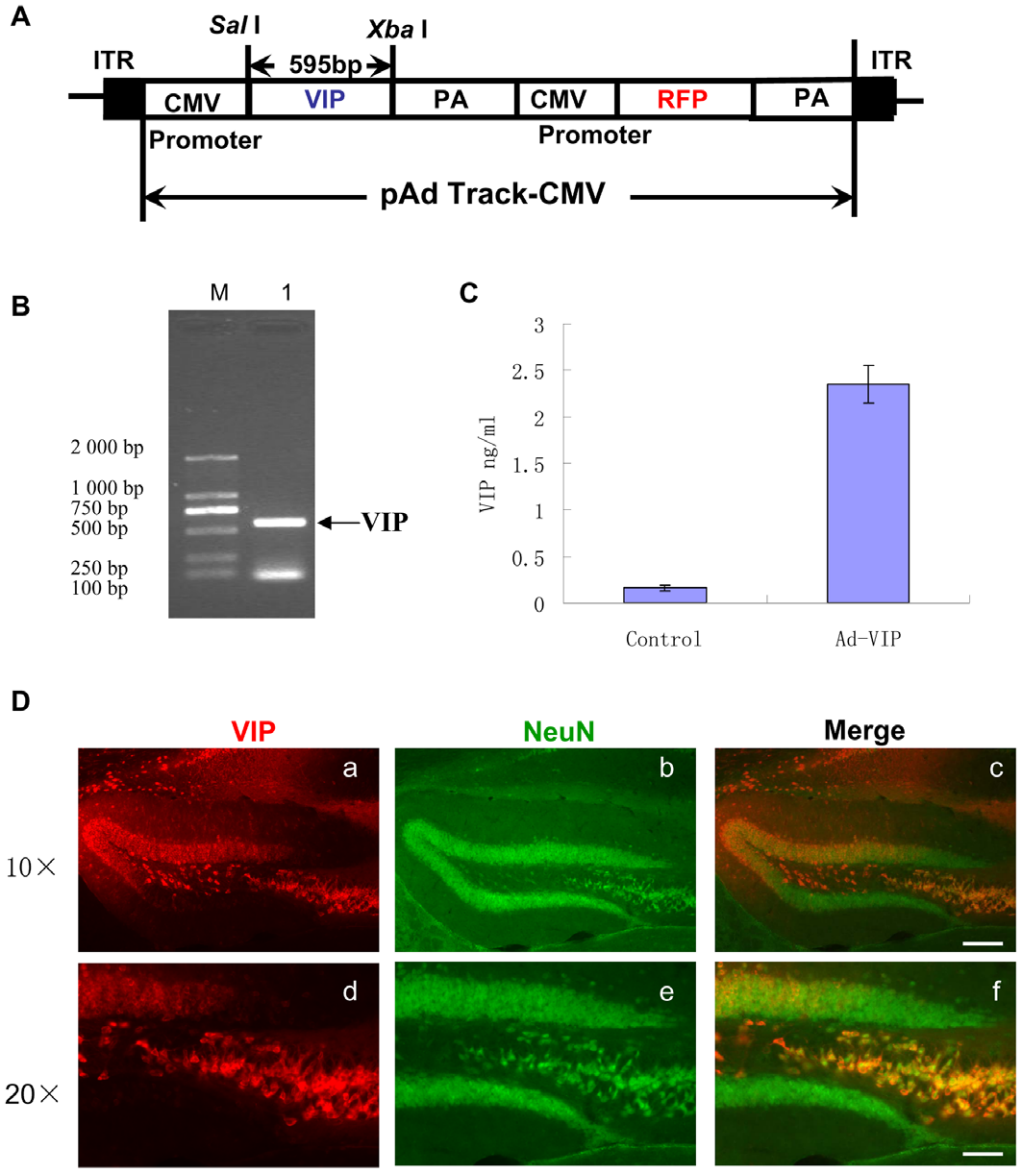
VIP highly expressed in the hippocampus NeuN positive cells after unilateral lateral ventricular injection of Ad-VIP. A: Schematic diagram of VIP adenovirus construction. B: Lane 1 shows the PCR product, corresponding to the mouse VIP cDNA (595 bp). Samples were run on a 2% agarose gel with a DNA ladder (left side). VIP cDNA was also verified by sequencing. C: VIP expression in rAd5CMVhVIP-infected cells. Culture media were assayed for VIP by an ELISA kit. Data shown are the total amount of VIP (ng) per 10^6^ infected 293 cells (Multiplicity of infection 320) per 24 h. The results are representative of five experiments with 3 replicates. D: Brain sections were prepared from TgAPPswe/PS1dE9 mice two months after Ad-VIP and subjected to double-immunofluorescence staining of VIP(red) and NeuN(green) using anti-VIP antibody and anti-NeuN antibody. Ad-VIP injected brain showing widespread, high expression of VIP in the hippocampus and somewhat less expression in the neocortex(a, d). NeuN staining showed with green fluorescence protein (b, e) and Merged picture showed(c,f). Scale bars 400 µm (a through c) and 200 µm (d through f).

We next determined whether VIP treatment could affect Aβ plaque formation and accumulation in a mouse model of Alzheimer's disease. Since exogenous VIP is rapidly degraded [27], we adopted viral vectors for sustained and local expression of a VIP transgene. We constructed a recombinant adenovirus vector encoding functional VIP, Ad-VIP (Fig. 4A), as described previously [28]. The infectious titer of the Ad-VIP vector was 1.0×10^11^ pfu/mL. Expression of VIP protein was determined in virus-infected 293 cells. Typically, 2×10^2^ particles of Ad-VIP vector/cell resulted in an almost 20-fold increase of VIP in the culture medium per 10^6^ infected 293 cells (Fig. 4C).

We then stereotaxically injected the recombinant fluorescence-labeled adenovirus Ad-VIP into the right lateral ventricle of 10 month old PS1/APPsw mice (expressing mutant APP and PS1). A control group of PS1/APPsw mice were injected with a blank recombinant adenovirus, Ad-Blank, or with PBS. After two months, mice were sacrificed and VIP expression was quantified by red fluorescence distribution and intensity. Widespread and intense fluorescence was observed in the dentate gyrus (DG). Combined staining of neuronal cell bodies with NeuN revealed that Ad-VIP could be efficiently expressed in DG granule and hilar neurons for as long as two months (Fig. 4D).

**Figure 5:**
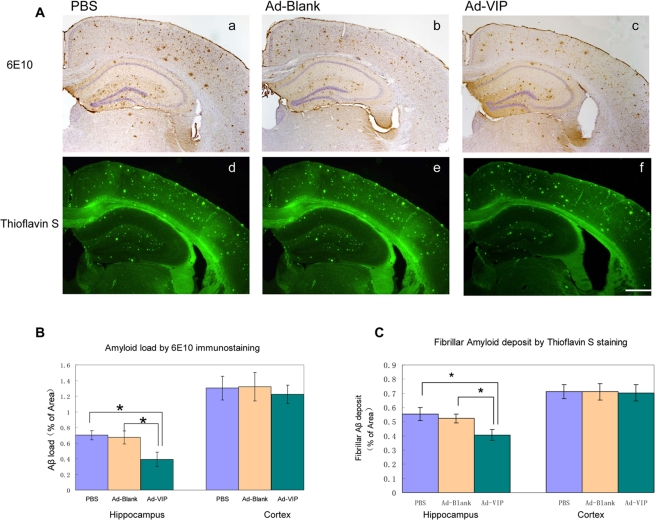
Adenovirus-mediated VIP expression reduces immunoreactive Aβ deposits and fibrillar Aβ deposits in the hippocampus but not in the neocortex. (a through h) Two months after Ad-VIP, Ad-Blank, or PBS injection, mice were terminated and Aβ deposits in the brain were visualized by immunohistochemistry and thioflavine S staining quantified by morphometric analysis. Diffuse and fibrillar Aβ deposits visualized by 6E10 antibody (a through c) and thioflavine S staining (d through f) in the cerebral cortex and hippocampus from mice injected with PBS (a and d), Ad-Blank (b and e) and Ad-VIP (c and f). The percentages of immunoreactive area for Aβ and thioflavine S fluorescent, respectively, are shown as bar graphs(g, h). The values shown are the mean± S.E.M. Scale bars 400 µm (a through f). ANOVA and post hoc testing revealed significant differences between different group. *P<0.05.

**Figure 6:**
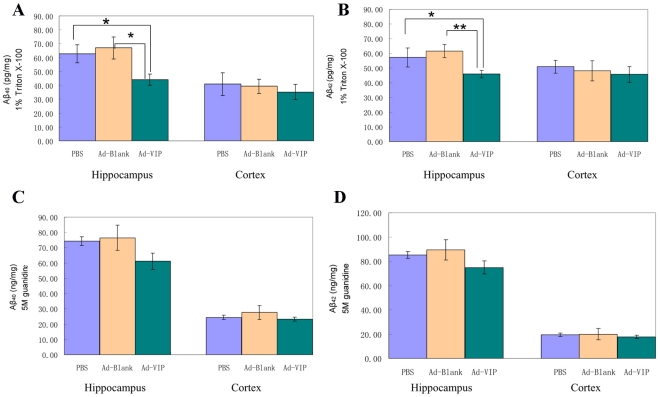
Adenovirus-mediated VIP expression reduces soluble Aβ_42_& Aβ_40_ in the hippocampus at 12 months of age. A, B: The buffer-soluble Aβ_42_ & Aβ_40_ contents in different treated Tg mice were quantified by ELISA. The results are shown as bar graphs (means±SEM pg/mg protein). *P<0.05. C: D: The buffer-insoluble Aβ_42_ contents in different treated Tg mice were quantified by ELISA. The results are shown as bar graphs (means±SEM ng/mg protein).

To determine the efficacy of adenovirus-mediated VIP expression in reducing Aβ load in the brain, diffuse and fibrillar Aβ deposits were detected by immunohistochemistry using the anti-Aβ 6E10 antibody and quantified by morphometry (Fig. 5A, a–c and 5B). The amyloid load was expressed as the percent area showing Aβ immunoreactivity. The average Aβ load in hippocampus was 0.39±0.09% in the Ad-VIP-injected group, significantly lower than in either the Ad-Blank group (0.65±0.15%) or the PBS-injected group (0.70±0.06%) (Fig. 6B). Thus, Ad-VIP injection reduced the Aβ load in the hippocampus by approximately 40% compared to the blank vector (*P* = 0.04, n = 8 for each group). In the neocortex, however, the average amyloid load in Ad-VIP-injected mice (1.25±0.10%) not significantly less than in Ad-blank-injected mice (1.34±0.17%) or PBS-injected mice (1.32±0.15%) (Ad-VIP vs. PBS, *P* = 0.66).

Fibrillar Aβ deposits were detected by thioflavin S staining and quantified by morphometric analysis (Fig. 5A, d–f and 5C). Again, the Aβ loads were quantified by the average percent area showing thioflavin S fluorescence. The average hippocampal Aβ load in Ad-VIP-injected mice (0.39±0.03%) was significantly lower than in Ad-blank-injected mice (0.53±0.03%) and PBS-injected mice (0.57±0.04%). Thus, Ad-VIP injection reduced thioflavin S staining in the hippocampus by approximately 25% compared to Ad-blank injection (*P* = 0.01, n = 8 for each group). In the neocortex, there was no difference in the Aβ load as measured by thioflavine S fluorescence between any of the mouse treatment groups (Fig. 5C).

We further determined the levels of buffer-soluble and in-soluble Aβ in the treatment groups by Aβ_40_- and Aβ_42_-specific sandwich ELISAs. In the hippocampus, the buffer-soluble Aβ_42_ content in Ad-VIP-injected mice (45.97±2.57 pg/mg protein) was significantly lower than in Ad-blank-injected mice (61.65±4.5 pg/mg protein) and PBS-injected mice (57.29±6.48 pg/mg protein) (Figure 6A). Injection of Ad-VIP reduced the buffer-soluble Aβ_42_ level in the hippocampus by approximately 27% compared to Ad-blank injection (*P* = 0.011, n = 7 for each group). The buffer-soluble Aβ_40_ content in Ad-VIP-injected transgenic mice (44.04±3.98 pg/mg protein, n = 7) was also significantly lower than in Ad-Blank-injected mice (66.90±7.86 pg/mg protein, n = 7, *P* = 0.03) (Fig. 6B). The buffer-insoluble Aβ_42_ and Aβ_40_ in Ad-VIP-injected mice (74.82±5.88 ng/mg protein and 61.15±5.36 ng/mg protein, respectively) was moderately reduced compared to the Ad-blank-injected mice (89.32±5.04 ng/mg protein, 76.50±8.27 ng/mg protein, respectively) and PBS-injected mice (85.19±5.31 ng/mg protein,74.24±2.92 ng/mg protein, respectively), but the difference did not reach statistical significance (Ad-VIP vs. Ad-Blank, *P* = 0.13 for insoluble Aβ_42_, P = 0.12 for insoluble Aβ_40_) (Fig. 6C, D).

In the neocortex, buffer-soluble Aβ_42_ in Ad-VIP-injected mice (45.71±5.41 pg/mg protein) was not significantly lower than in Ad-blank-injected mice (48.18±6.91 pg/mg protein) or PBS-injected mice (50.94±4.43 pg/mg protein) (Ad-VIP vs. PBS, *P* = 0.47). There was no difference in the insoluble Aβ_42_ or insoluble Aβ_40_ between any treatment groups (Fig. 6C).

### Ad-VIP injection activated CD11b-immunoreactive microglial in the hippocampus, but not CD11c- or CD45-immunoreactive microglia

**Figure 7:**
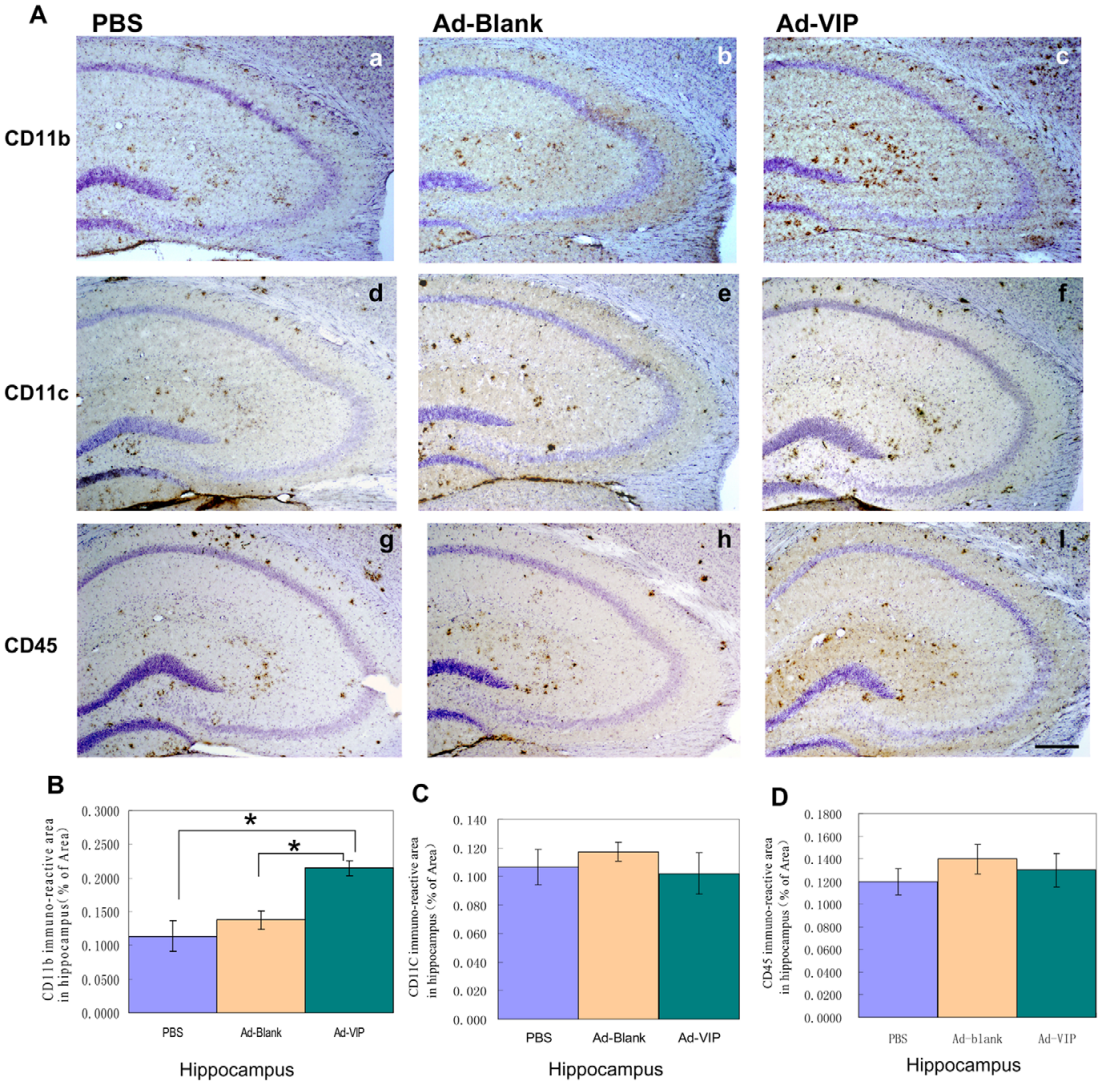
CD11b but not CD11c or CD45 immunoreactive microglia significantly activated in the hippocampus of Ad-VIP injected mice. A: (**a**–**f**) Brain sections from mice injected with PBS (**a**), Ad-Blank (**b**) and Ad-VIP (**c**) were stained with anti-CD11b antibody or anti-CD11c(**d,e,f**) antibody or anti-CD45(**g,h,i**) antibody Scale bars are 200 µm in **a** through **i**. B: CD11b immunoreactive quantified by morphometric analysis. CD11b immunoreactive area percentages in the hippocampus are shown as a bar graph (means ± S.E.M.) *P<0.05. C: CD11c immunoreactive quantified by morphometric analysis. CD11c immunoreactive area percentages in the hippocampus are shown as a bar graph (means ± S.E.M.). D: CD45 immunoreactive quantified by morphometric analysis. CD1145 immunoreactive area percentages in the hippocampus are shown as a bar graph (means ± S.E.M.).

The role of microglial activation in Aβ plaque reduction in PS1/APPsw mice was investigated by immunofluorescence labeling (Fig. 7). The average CD11b immunoreactive area in the hippocampus was 0.21±0.03% in Ad-VIP-injected mice, 0.14±0.03% in Ad-Blank-injected mice, and 0.11±0.03% in mice injected with PBS (Fig. 7A, a–c, 7B). Thus, Ad-VIP injection enhanced the accumulation of CD11b-immunoreactive microglial cells in the hippocampus by approximately 50% compared to Ad-Blank injection (*P* = 0.003, n = 8 for each group). However, the numbers of CD11c-immunoreactive microglial cells were not affected significantly as indicated by the lack of change in immunoreactive areas (0.10±0.012%, 0.12±0.007% and 0.10±0.014% for mice injected with Ad-VIP, Ad-Blank, and PBS, respectively; Fig. 7A,d–f, 7C). We also investigated CD45-immunoreactive microglial cells to investigate the activation stage of microglia in these transgenic mice. The average CD45 immunoreactive areas in the hippocampus were not significantly different between injection groups (0.13±0.013%, 0.14±0.009% and 0.12±0.012% for mice injected with Ad-VIP, Ad-Blank, and PBS, respectively; Fig. 7A g–i, 7D), consistent with the lack of change in CD11c staining.

### Recombinant adenovirus-mediated VIP brain delivery does not increase gliosis and apoptosis in APP/PS1 transgenic mice

**Figure 8:**
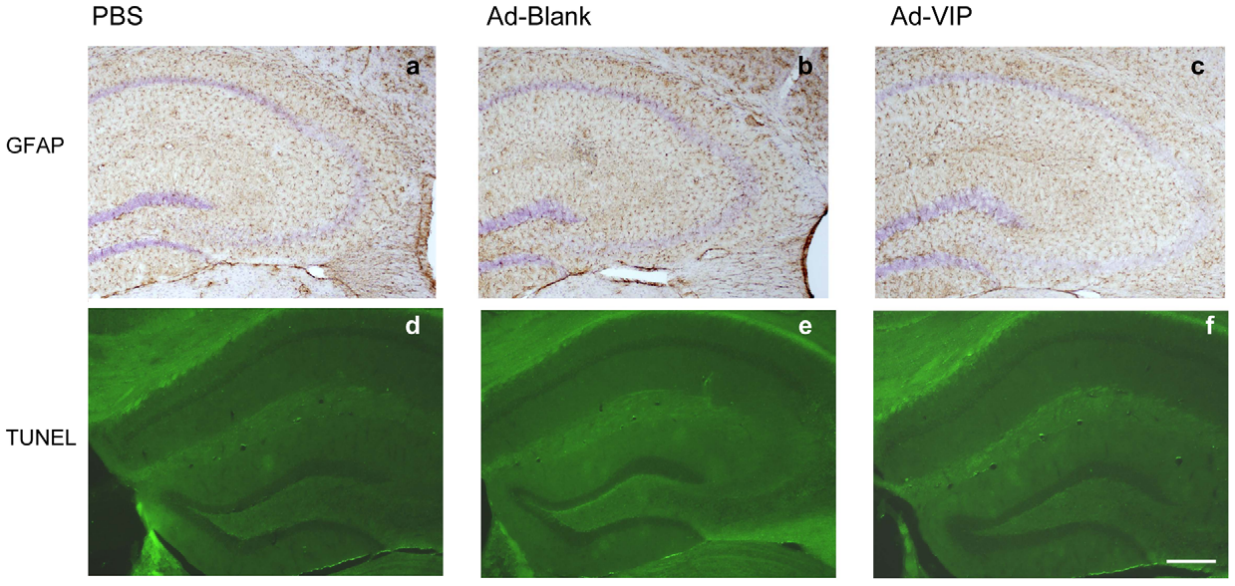
Recombinant adenovirus-mediated VIP brain delivery does not increase gliosis and apoptosis. (**a**–**f**) Brain sections from mice injected with PBS (**a**), Ad-Blank (**b**) and Ad-VIP (**c**) were stained with anti-GFAP antibody and Apoptotic cells identified by Tunel staining(**d, e, f**). Scale bars are 200 µm in **a** through **f**.

High levels of VIP expression activated microglial cells, which may also induce the secretion of neurotoxins (cytokines and NO), leading to gliosis and apoptosis. To assess the cytotoxic potential associated with recombinant adenovirus-mediated overexpression of VIP, brain sections were subjected to Glial fibrillary acidic protein (GFAP) immunohistochemistry and Terminal (TdT)-mediated dUTP-biotin nick end labeling (TUNEL) assay. The average GFAP immunoreactive area in the hippocampus was 4.41±0.51% in the Ad-VIP group, 5.8±0.53% in the Ad-Blank group, and 5.2±0.58% in the PBS group, indicating that VIP overexpression slightly reduced gliosis compared to Ad-blank treatment, but not significantly (P = 0.099, n = 8 for each group) (Fig. 8Aa–c). No apoptotic cell death was detected by TUNEL staining in the brains injected with Ad-VIP (Figure 8f), suggesting that adenovirus-mediated VIP overexpression in the hippocampus does not induce neuronal or glial apoptosis.

## Discussion

The principal pathological hallmark of Alzheimer's disease is the extracellular deposition of fibrillar Aβ and its compaction into senile plaques surrounded by activated microglia and astrocytes (reactive gliosis). Microglial activation is a “double-edged” sword in that these cells release proinflammatory cytokines, pro-apoptotic factors and reactive oxygen and nitrogen species in addition to protective neurotrophins. In addition, microglial phagocytosis can protect neurons from toxic substances, such as aggregated Aβ. Regulating microglial phenotype to enhance protective mechanisms (neurotrophin release and phagocytosis) while suppressing neurotoxic effects could be the basis for novel therapeutic strategies. Consistent with the role of neuropeptides as critical modulators of the immune response in the CNS [29], we demonstrated that VIP regulated microglial activation by fibrilized-Aβ_42_. Indeed, VIP significantly promoted microglia phagocytosis of Aβ_1–42_ and attenuated cerebral amyloidosis in a transgenic mouse model of AD.

Previous studies examining the effects of VIP on phagocytosis in macrophages yielded conflicting results. Delgado et al concluded that a common denominator in these studies was that VIP stimulated freshly isolated resting macrophages but inhibited prestimulated macrophages. Therefore, the differences in the reported studies could be due to the dominant cell population. In addition, the stimulatory actions of VIP (whether for phagocytosis, adherence and migration, or reactive oxygen production) appear to be associated with PKC activity. Our experiment suggested that the effect of VIP on phagocytosis of microglial cells trend to be stimulatory and associated with PKC activity, Myristoylated protein kinase C peptide inhibitor, which inhibit PKC pathway could efficiently block this effect.

Injection of Ad-VIP into the lateral ventricle of PS1/APP transgenic mouse induced significant VIP overexpression and attenuated immunoreactive Aβ deposits and fibrillar Aβ deposits in the hippocampus two months after injection. In contrast, Aβ levels in the cortex were not altered. These restricted of VIP effects may be due to the limited expression of VIP outside the hippocampus. To study the cellular mechanism of Aβ clearance, we measured changes in microglial and astrocytic immunoactivity. Surprisingly, we found that CD11b-immunoreactive microglial cells accumulated in the Ad-VIP-injected PS1/APP transgenic mouse hippocampus relative to Ad-blank- or PBS-injected PS1/APP transgenic mice, while CD11c-immunoreactive microglial cells were not significantly altered by Ad-VIP-mediated VIP overexpression. The activation of CD11b-positive microglial cells may be due to VIP-induced activation of cAMP and PI-3K signaling pathways [30]. A previous study demonstrated that CD11b-positive microglial cells trend to have enhanced phagocytotic activity while CD11c-positive microglial cells trend to initiate antigen presentation in response to cytokine secretion [6]. Recently, Nada Choucair-Jaafar reported that an antibody against CD11b reduced the uptake of artificial amyloid deposits by microglia, further indicating that CD11b+ microglia are involved in Aβ clearance [31]. These results may also partially explain why the Aβ load was reduced in the hippocampus of AD-VIP-treated mice. In contrast, adenovirus-mediated VIP brain delivery did not increase GFAP expression by astrocytes or neurocellular TUNEL staining, indicating that VIP overexpression did not induce astrogliosis or apoptosis in APP/PS1 transgenic mice. Thus, recombinant adenovirus Ad-VIP injections may be a feasible and safe treatment to promote the protective effects of microglial cells without deleterious side effects.

In addition to promoting Aβ accumulation, VIP also blocked Aβ-induced microglial TNF-α and NO secretion, consistent with previous studies [24]. As a central inflammatory mediator, TNF-α plays a key role in neuronal death mediated by activated microglia. Elevated TNF-α levels were detected in the serum and CSF of AD patients, as well as in cortical and glial cell cultures after exposure to Aβ. Furthermore, high levels of TNF-α induced apoptosis of cortical neurons by activating the neuronal TNF-α receptor [32], [33]. Activated microglia also release NO, which can form highly neurotoxic NO_2_^−^. Weldon et al. demonstrated that NO secreted by activated microglia could kill culture human fetal neurons by apoptosis [34]. Many immune activators, such as LPS, can promote microglial phagocytosis of Aβ and reduce the Aβ load in transgenic mice [35]. However, most of these agents can not inhibit concomitant microglial TNF-α or NO secretion, and may even increase TNF-α and NO secretion, which abate the possible clinical application. Recently, Delgado et al showed that VIP efficiently inhibited the secretion of cytokines associated with microglial activation induced by Aβ [24]. Consider with Our study that the presence of VIP resulted in a significant promotion of microglial phagocytosis of fibrilized-Aβ, hinting that VIP may be an ideal treatment for AD.

Studies addressing the neuroprotective efficacy of VIP in a Parkinson's disease model revealed that VIP could prevent MPTP-induced nigrostriatal dopaminergic neuronal death by blocking microglial activation [36]. Furthermore, Gozes et,al found that the Aβ-induced cortical neuronal death was completely prevented by co-treatment with 0.1 pM [St-Nle7] VIP and that St-Nle-VIP injected intracerebroventricularly or delivered intranasally prevented impairments in spatial learning and memory associated with cholinergic blockade in a rat cholinergic deficits model [37]. Neuroinflammation may be a pathogenic mechanism common to several neurodegenerative diseases, so VIP may be widely applicable as a neuroprotective agent by suppressing the pathological activities of activated microglia.

In summary, our results demonstrated that VIP promoted microglia phagocytosis of Aβ and suppressed the Aβ-induced release of microglial neurotoxins. Furthermore, constitutive overexpression of VIP in the hippocampus reduced inflammation and attenuated amyloidosis in a transgenic mouse model of AD. Modulation of Aβ-induced microglia activation and microglial phenotype by delivery of VIP into the brain may be a novel approach for the treatment of Alzheimer's diseases.

## Materials and Methods

### Reagents

β-Amyloid peptide(Aβ)1–42, Cy3 conjugated Aβ1–42 peptides were purchased from Biosource International (Camarillo, CA). The peptides were pre-aggregated for 24 h at 37°C in complete medium. Vasoactive Intestinal Peptide was obtained from Sigma(St.Louis, MO) and dissolved in 0.01 M phosphate-buffered saline (PBS) to a stock concentration of 10^−6^ M. Recombined mouse IFN-γ were purchased from BD Pharmigen(San Diego,CA).

### Cell Cultures

The murine microglial cell line (N9) was kindly provided by Dr. Ji Ming Wang (Center for Cancer Research, National Cancer Institute, Frederick, USA) and were grown in RPMI 1640 medium supplemented with 5% fetal calf serum, 2 mM glutamine,100 U/mL penicillin, 0.1 g/mL streptomycin and 0.05 mM 2-mercaptoethanol.

Mouse primary microglial cells culture was prepared as described previously [15]. Briefly, cerebral cortices from newborn C57/BL6 mice (1–2 days old) were isolated under sterile conditions and were kept at 4°C. Cells were mechanically dissociated and plated in 75-cm^2^ flasks, added RPMI 1640 medium supplemented with 5% fetal calf serum, 2 mM glutamine,100 U/ml penicillin, 0.1 ug/ml streptomycin, and 0.05 mM 2-mercaptoethanol. Primary cultures were kept for 14 days so that only glial cells remained and microglial cells were isolated by shaking flasks at 200 rpm in a Lab-Line incubator-shaker. More than 98% of these glial cells stained positively for CD11b (BD Pharmigen, San Diego, CA). All animals were provided by animal center of Third Military Medical University and all experiments were in compliance with protocols(IACUC: 06512) approved by the Third Military Medical University Institutional Animal Care and Use Committee.

### Microglia Aβ phagocytosis assays

Primary mouse microglia were seeded at 1×10^5^ cells/well (n = 6 for each condition) in 24-well tissue culture plates containing 0.5 mL of complete RPMI 1640 medium. These cells were treated for 2 h with “aged” Aβ_1–42_ conjugated with Cy3 (Biosource International; 500 nM pre-aggregated for 24 h at 37°C in complete medium). In the presence of Cy3-Aβ_1–42_, microglial cells were then treated with VIP, LPS. Some of these cells were treated with PMA (30 nM), Forskolin (10 µM), Myristoylated protein kinase C peptide (50 µM) combined with VIP, PKA inhibitor fragment 14–22 myristoylated trifluoroacetate salt (PKA Inhibitor) (5 µM)(Sigma, St.Louis, MO) combined with VIP in the presence of Cy3-Aβ_1–42_ for 2 h. Microglial cells were then rinsed three times in complete medium, and the media were exchanged with fresh complete medium for 10 minutes to allow for removal of non-incorporated Aβ_1–42_ and promote concentration of Aβ_1–42_ into phagosomes. Extracellular and cell-associated Cy3-Aβ_1–42_ were quantified using an MSF (SpectraMaxH, Molecular Devices) with an emission wavelength of 590 nm and an excitation wavelength of 543 nm.

A standard curve from 0 to 600 nM of Cy3-Aβ was run for each plate. Total cellular proteins were quantified using the Micro BCA Protein Assay (Pierce, Rockford, IL). The mean fluorescence values for each sample at 37°C and 4°C at the 2 h point were determined by fluorometric analysis. Relative fold change values were calculated as: mean fluorescence value for each sample at 37°C/mean fluorescence value for each sample at 4°C. In this manner, both extracellular and cellassociated Cy3-Aβ_1–42_ were quantified. Considering nonspecific adherence of Aβ to the plastic surface of culture plates, an additional control without cells was carried out through all of experiments above. An incubation time of less than 4 h did not change the amount of Aβ_1–42_ peptide detected in the supernatant. In order to determine the extent to which cell death might have influenced the phagocytic activity in the various treatment groups, we performed the LDH assay on the relevant supernatant. Data showed that there was no significant cell death occurring over the 3 h time frame in any of the treatment groups.

### Fluorescence microscope examination

Microglial cells (wild-type primary cultured microglial cells or N9) phagocytosis of fibrillar Aβ_1–42_βpeptides was carried out similarly to previously described protocols [38], Microglial cells were cultured at 2×10^5^/well in 12-well tissue-culture plates with glass inserts for fluorescence microscopy. On the following day, microglia cells were treated with Cy3-conjugated Aβ1–42 (500 nM) in the presence or absence of VIP (10^−8^ M) for a different phage of time. As a positive control, microglial cells were treated with Cy3-conjugated Aβ_1–42_ (500 nM) combined with LPS(100 ng/mL, Sigma). In parallel dishes, microglial cells were incubated with Cy3-conjugated Aβ_1–42_ under the same treatment conditions as described above, except that they were incubated at 4°C to control for nonspecifically cellular association of Cy3-Aβ_1–42_. Microglial cells were then rinsed 3 times in Cy3-Aβ_1–42_-free complete medium and the media was exchanged with fresh Cy3-Aβ_1–42_-free complete medium for 10 min both to allow for removal of non-incorporated Cy3-Aβ_1–42_ and to promote concentration of the Cy3-Aβ_1–42_ peptide into phagosomes. This medium was withdrawn, and microglia were rinsed 3 times with ice-cold PBS. For fluorescence microscope examination, these Microglial cells cells will be washed 5 times with ice-cold PBS to remove extracellular Aβ and fixed in 4% paraformaldehyde(PFA) diluted in PBS, After three successive rinses in TBS, microglia nuclei were detected by incubation with DAPI for 10 min and finally mounted with fluorescence mounting media containing Slow Fade antifading reagent (Molecular Probes, Eugene, OR), and then viewed under a BX61 microscope (Olympus, Tokyo, Japan) equipped with a digital camera. The data was digitized using an attached Image Pro Plus imaging system (MediaCybernetics).

### Cytokine analysis

Microglial cells (N9 or wild-type primary cultured microglial cells) were plated in 24-well tissue-culture plates (Costar, Cambridge, MA) at 1×10^5^ cells per well and stimulated for 24 hr with either Aβ_1–42_ (2.5 µM) or a dose range of IFN-γ (10 U/mL, 200 U/mL)/Aβ_1–42_ (2.5 µM) in the presence or absence of VIP (10^−8^ M). Cell-free supernatants were collected and stored at −70°C until analysis. TNF-α cytokine and NO levels in the supernatants were examined using specific enzyme-linked immunosorbent assay (ELISA) kits (R&D Systems) or Griess Reagent Kit for Nitrite Determination (Promega) in strict accordance to the manufacturer's protocols. The results of TNF-α cytokine are shown as mean picograms of each cytokine per milliliter (±SD)and The results of NO represent as mean µM(±SD).

### Recombinant adenovirus preparation

Recombinant adenovirus pAdeasy-VIP (1×10^11^ PFU/ml) and blank adenovirus(1×10^11^ PFU/ml) was generated by Min Song according to the methods [28] (Department of medical genetics, Third Military Medical University), Briefly, VIP was amplified by RT-PCR and cloned into a shuttle pAd Track-CMV vector with Red florensence Protein (RFP) tag. This plasmid was linearized by digesting with restriction endonuclease PmeI and subsequently cotransformed into Escherichia coli BJ5183 cells with an adenoviral backbone pAdEasy-1 plasmid. Recombinants were selected by kanamycin resistance. Finally, recombinants were transfected into HEK293 cells. Recombinant adenoviruses were generated within 7–10 d. then purified with Sartbind Q5 column purification system(Sartorius stedim biotech, Aubagne Cedex, France). The titers of Adenovirus were determined by the quantitative fluorescence assay as described previously [39]. A control null Adenovirus (Ad-blank) which only express Red florensence Protein was similarly prepared. VIP protein expression was determined by ELISA kit (Peninsula Laboratories, San Carlos, CA, USA), Typically, 2×10^2^ particles of Ad-VIP vector/cell resulted in almostly 20-fold increase over background in the culture medium per 10^6^ infected 293 cells.

### Experimental animals and stereotaxic injection of Ad-VIP

Tg(APPswe, PSEN1de9) mice (line B6C3-85Dbo/J) were purchased from Jackson Laboratory (Bar Harbor, ME). The transgenic mice express chimeric mouse/human APP with the double mutations (K670N and M671L) and human PS1 with a deletion of exon 9 found in familial AD patients. Animals were housed and maintained at the College of Medicine Animal Facility of the Third Military Medical University. A total of forty-five 10 months old Tg PS1/APPsw mice were randomly assigned to 3 treatment groups in such a manner as there was no significant intergroup difference in body weight: PBS, Ad-blank, Ad-VIP (n = 15 for each group, 8 mice for histochemical analyses and 7 mice for protein analysis).

The intra-lateral ventricle injections surgery procedure was according with Giovanni DiCarlo described previously [35]. Briefly, the mice were weighed, anesthetized with pentobarbital and placed in a stereotaxic apparatus (51603 dual manipulator lab standard, Stoelting, Wood Dale, IL). A midsagittal incision was made to expose the cranium, A hole in the skull was made by a drill 0.5 mm posterior to the bregma and 1.0 mm right to the midline. A 10 µl injection of PBS or recombinant adenovirus Ad-VIP (1×10^11^ PFU/ml) or blank adenovirus, Ad-blank(1×10^11^ PFU/ml) was made using a 10 µl syringe (Hamilton, Reno, Nevada) was injected unilaterally into the right ventricle at the depth of 2 mm at a rate of 1 µl/min. After allowing the needle to remain in place for 5 min, the needle was slowly raised at a rate of 0.1 cm/min. Two months after the injection, the experimental animals were terminated to determine the therapeutic effects of the treatment. All animal protocols (IACUC: 06513) used for this study were prospectively reviewed and approved by the Institutional Animal Care and Use Committee of Third Military Medical University Institutional Animal Care and Use Committee.

### Immunohistochemical and histochemical analyses

Mice were deeply anesthetized with pentobarbital and cardinally perfused with cold PBS followed by 4% paraformaldehyde. The brain was quickly removed and fixed in 4% paraformaldehyde for 16 h.

The brains were then stored overnight in 30% sucrose in 0.1 M PBS and frozen in Tissue-Teck optimal cutting temperature compound. coronal sections (35 µm thick) of the brains were cut on a freezing-stage cryotome and kept in 0.1 M PBS at 4°C. Sections were subjected to immunohistochemical, histochemical, and TUNEL staining. For immunohistocheimstry, free floating immunohistochemistry was performed using avidin-biotin immunoperoxidase method (Vector, Burlingame, CA). Endogenous peroxidase was eliminated by treatment with 1% H_2_O_2_/10% methanol Tris-buffered saline (TBS) for 60 min at room temperature. After washing with 0.1 M Tirs buffer (pH 7.5) and 0.1 M TBS (pH 7.4), sections were blocked with 5% normal serum (from the same animal species in which the secondary antibody was made) in 0.1 M TBS with 0.5% triton-X-100 (TBS-T) for 60 min at room temperature to prevent non specific protein binding. The sections were then incubated with primary antibodies as described above and 6E10 for detection of Aβ in 2% serum in TBS-T for 18–48 h at 4°C. The sections were rinsed in 0.1 M TBS and incubated with biotinylated secondary antibodies in 2% serum TBS-T for 2 h at room temperature. Finally, the avidin biotin peroxidase method using 3, 3′-diaminobenzidine as a substrate (Vector, Burlingame, CA) was performed according to manufacturer's protocol. For the negative control, slides were processed without primary antibody. Some sections were counterstained with hematoxylin. For thioflavineS staining, tissue sections were stained in 1% thioflavine S (Sigma) and rinsed with 70% ethanol. After washing with H_2_O, the sections were mounted in 75% glycerol in H_2_O. Neuroinflammation was detected by staining the brain sections with rat anti-mouse CD11b, rat anti-mouse CD11c, rat anti-mouse CD45, rabbit anti-mouse GFAP antibodies BD Pharmigen(San Diego, CA).

### Immunofluorescence

Free-floating brain sections were used to analyze Adenovirus mediated VIP expression, Immunofluorescence detection and staining for VIP and NeuN was performed using the anti-NeuN antibody (1∶400). Sections were incubated in anti-NeuN antibodies overnight at 4 C, rinsed in PBS, and incubated for 1 h in fluorescently labeled secondary antibodies. Sections were washed three times with PBS. To assess nonspecific labeling, a negative control sample was processed using the same protocol but with the primary antibody omitted. Cells were counted with a BX61 microscope (Olympus, Tokyo, Japan) equipped with a digital camera.

### Quantification of buffer soluble and in-soluble brain Aβ by ELISA

the treated Mice(n = 7 for each group) were deeply anesthetized with pentobarbital and cardinally perfused with cold PBS. The isolated hippocampus and cortex were weighted and then placed in ice-cold lysis buffer, sonicated on ice for ∼3 min, allowed to stand for 15 min at 4°C, and centrifuged at 15,000 rpm for 15 min. Levels of buffer-soluble and insoluble ***Aβ*** in the hippocampus were determined by the ***Aβ***_42_ and ***Aβ***_40_ ELISA kits (Invitrogen, Carlsbad, CA) according to the manufacturer's protocol. Levels of buffer-soluble *Aβ* were expressed as mean pg/mg of total protein in tissue lysate standard error. Levels of buffer-insoluble *Aβ* were expressed as mean ng/mg of wet tissue weight standard error.

### TUNEL assay to detect apoptosis

To investigate cytotoxicity possibly associated with the therapeutic modalities, brain sections were subjected to Terminal (TdT)-mediated dUTP-biotin nick end labeling (TUNEL) assay using the In situ Cell Death Detection Kit (Roche Biochemicals, Indianapolis, IN) according to the manufacturer's protocol. Slides were analyzed using a BX61 microscope (Olympus, Tokyo, Japan) equipped with a digital camera.

### Image analysis

Quantitative image analysis was performed for amyloid deposits and reactive/activated glial cells immunohistochemistry in Tg PS1/APPsw mice injected with Recombinant adenovirus Ad-VIP or blank adenovirus or PBS. Images were performed using an Olympus BX61 automated microscope, Olympus Fluoview system and the Image Pro Plus v5 image analysis software (Media Cybernetics, Silver Spring, MD). Briefly, images of five 35 µm sections each separated by an approximately 400 µm interval, starting at 1.3 mm posterior to the bregma to caudal, from each mouse were analyzed. Both neocortex and hippocampus were found in all the brain sections and analyzed separately. Stained areas were expressed as a percentage of total neocortex or hippocampus, respectively. a threshold optical density was obtained that discriminated staining from background. Manual editing of each field was used to eliminate artifacts. Data are reported as a percentage of immuno-labeled area captured (positive pixels) divided by the full area captured (total pixels). Quantitative image analysis was performed by a single examiner (T.M.) blinded to sample identities. Data were expressed as mean ± standard error of the mean (SEM) as a bar graph.

### Statistical analysis

All data were normally distributed; therefore, in instances of single mean comparisons, Levene's test for equality of variances followed by a *t* test for independent samples was used to assess significance. In instances of multiple mean comparisons, ANOVA was used, followed by post hoc comparison using Bonferonni's method. α Levels were set at 0.05 for all analyses. The statistical package for the social sciences release 10.0.5 (SPSS, Chicago, IL) was used for all data analyses.
